# QSPR analysis using stress sum based topological indices for predicting physicochemical properties of Hodgkin lymphoma drugs

**DOI:** 10.3389/fchem.2026.1936029

**Published:** 2026-09-15

**Authors:** A Sivaranjani, K Bharani Dharan, S Radha

**Affiliations:** School of Advanced Sciences, Vellore Institute of Technology, Chennai, India

**Keywords:** leave one out cross validation, linear regression, molecular graph, QSPR analysis, stress sum based topological indices

## Abstract

Molecular graph theory has been widely used to represent the structure of chemical compounds and to study their physicochemical properties. In this work, we introduce a set of stress sum-based indices and examine their usefulness in describing the structural characteristics of drugs used in Hodgkin lymphoma treatment. Eight stress sum-based indices were calculated for thirteen selected drug molecules. Their relationships with seven physicochemical properties, namely boiling point, complexity, molecular weight, molar refraction, polar surface area, polarizability, and heavy atom count, were examined using Quantitative Structure–Property Relationship (QSPR) analysis. The correlation analysis of various indices supports the fact that the stress sum-based indices are efficient to capture the pertinent structural features of drug molecules and their application as molecular descriptors in QSPR studies. Additionally, these indices provide meaningful insights into the structural and physicochemical properties of drugs associated with Hodgkin lymphoma treatment. The findings of this study show promise for practical applications for drug design, structural optimization, and the repurposing of existing therapeutic compounds in the broader efforts of cancer pharmacology research.

## 1 Introduction

Hodgkin lymphoma (HL) is a distinct B-cell malignancy with characteristic pathological, clinical, and therapeutic features. Although it is relatively rare, HL represents a significant proportion of lymphomas diagnosed worldwide (Ansell, 2015). Advances in diagnostic methods and risk-adapted treatment strategies have considerably improved the survival of patients with HL. Nevertheless, the disease continues to present clinical challenges, particularly in older patients and in cases of relapsed or refractory disease (Barrett and Collins, 2022; Bosch-Schips et al., 2022; Brice et al., 2021; Fylenko et al., 2025; Maco et al., 2022; Ullah et al., 2022; Wilson et al., 2023). Molecular approaches, including circulating tumor DNA (ctDNA), are increasingly being used for diagnosis, disease monitoring, and prediction of relapse (Maco et al., 2022). These developments emphasize the need for continued investigation of the molecular and physicochemical characteristics of therapeutic compounds used in HL.

The treatment of HL involves a combination of conventional chemotherapeutic agents and newer targeted drugs. Alkylating agents such as *Carmustine* (Wheeler et al., 1990), *Cyclophosphamide* (Wheeler et al., 1990), *Dacarbazine* (Lee et al., 2025), *Lomustine* (Proctor et al., 2010), *Chlorambucil* (Proctor et al., 2010), and *Procarbazine* (Geurts et al., 2023) have been used extensively in the treatment of HL. Other important therapeutic agents include the anthracycline *Doxorubicin* (Lee et al., 2025), the vinca alkaloid *Vinblastine* (Proctor et al., 2010), and the antibiotic *Bleomycin* (Proctor et al., 2010), which are components of the widely used ABVD regimen. Drugs such as *Dexamethasone* (Proctor et al., 2010), *Etoposide* (Wheeler et al., 1990), and *Prednisone* (Hall et al., 1967) are also used in different treatment settings. In addition, the introduction of targeted agents such as *Pemigatinib* (Hoy, 2020) reflects the increasing role of molecularly directed therapies. The structural diversity of these compounds provides a suitable setting for studying relationships between molecular structure and physicochemical properties using mathematical and computational approaches.

Molecular graph theory provides a convenient mathematical framework for representing chemical compounds as graphs, where atoms are represented by vertices and chemical bonds by edges. One important class of graph-theoretical descriptors used in chemical graph theory is the class of *topological indices*. These numerical descriptors transform structural information contained in a molecular graph into values that can be related to experimentally measured molecular properties. Classical degree-based and distance-based indices, including the Wiener, Randić, and atom–bond connectivity (ABC) indices, have been widely used for this purpose (Estrada et al., 1998; Randić, 2002; Wiener, 1947). Several other descriptors, such as the Zagreb indices (Gutman and Trinajstić, 1973), forgotten index (Furtula and Gutman, 2015), geometric–arithmetic index (Yuan et al., 2010), inverse sum indeg index (Pattabiraman, 2018), Shilpa–Shanmukha index (Zhao et al., 2021), and Sombor index (Gutman, 2021), have subsequently been introduced and investigated. Their successful application to different classes of pharmaceutical and chemical compounds demonstrates the usefulness of topological indices in relating molecular structure to physicochemical properties (Arockiaraj et al., 2025a; Arockiaraj et al., 2025b; Furtula and Gutman, 2015; Hayat et al., 2018; Kansal et al., 2023; Manickan and Desikan, 2024; Rao et al., 2024; Rauf et al., 2023a; Shanmukha et al., 2020; Shanmukha et al., 2022; Zaman et al., 2024; Zhong et al., 2021).

Among the structural descriptors developed from graph-theoretical concepts, stress-based measures provide information related to the occurrence of vertices and edges on shortest paths. The concept of stress centrality was introduced by Shimbel in 1953 (Shimbel, 1953), where the stress of a vertex is associated with the number of shortest paths passing through that vertex. More recently, stress-related descriptors have been studied in chemical graph theory, including the stress of vertices and the square root stress-sum index (Bhargava et al., 2023; Mahesh et al., 2021). Stress indices have also been evaluated for several graph families, such as complete bipartite graphs, paths, cycles, trees, windmill graphs, and wheels (Niveditha et al., 2025). These developments indicate that stress-based quantities can provide structural information that is different from that obtained solely from vertex degrees or distances.

Quantitative Structure–Property Relationship (QSPR) modeling provides a useful approach for investigating the relationship between molecular structure and measurable physicochemical properties. In QSPR studies, molecular descriptors such as topological indices are used as independent variables to develop mathematical relationships with selected molecular properties. Such models have been applied to a wide range of chemical and pharmaceutical compounds. Roy’s work on QSAR/QSPR modeling provides a theoretical basis for the use of molecular descriptors in drug discovery and related applications (Roy, 2017). In particular, graph-theoretical descriptors have been used in QSPR studies of antiepileptic drugs (Huilgol et al., 2022), COVID-19 drugs (Rauf et al., 2023b), biodegradable compounds (Lee and Min, 2022), alkanes (Chamua et al., 2024), and other pharmaceutical datasets (Hosamani et al., 2017). Similar studies have considered anticancer (Rao et al., 2024), antibacterial (Rao et al., 2024), lung cancer (Arockiaraj et al., 2025a), breast cancer (Rauf et al., 2023a; Shanmukha et al., 2022), skin cancer (Arockiaraj et al., 2025b), and blood cancer drugs (Zaman et al., 2024). These studies demonstrate that suitable graph-based descriptors can provide useful information about physicochemical properties and can therefore support the analysis of molecular structure in drug-related research.

Despite the extensive use of classical topological indices in QSPR studies, the application of stress-based analogues of degree-based indices to pharmaceutical compounds has received comparatively less attention. In the present work, we address this gap by defining eight *stress sum-based topological indices*, obtained by incorporating stress values into the framework of degree-based molecular descriptors. These indices combine information on shortest-path participation with structural information traditionally captured by degree-based descriptors. The proposed indices are calculated for thirteen drugs associated with HL, namely *Carmustine, Cyclophosphamide, Dacarbazine, Lomustine, Chlorambucil, Procarbazine, Pemigatinib, Doxorubicin, Vinblastine, Bleomycin, Dexamethasone, Etoposide, and Prednisone*. Their relationships with seven physicochemical properties, namely boiling point, complexity, molecular weight, molar refraction, polar surface area, polarizability, and heavy atom count, are examined using QSPR analysis. The obtained results are used to assess the effectiveness of the proposed stress sum-based indices as molecular descriptors for HL-related drug molecules and to explore their possible usefulness in drug design, optimization, and repurposing.

## 2 Materials and methods

Let G denote the hydrogen-suppressed molecular graph representation of a Hodgkin lymphoma drug, where V(G) represents the set of vertices (atoms) and E(G) represents the set of edges (chemical bonds) in the molecular structure. The order and size of the graph G are denoted by n and m respectively. For two vertices u,v∈V(G) , the edge joining them is denoted by uv . The *stress* of a vertex u , denoted by st(u) , is defined as the number of shortest paths that pass through u (Shimbel, 1953). The *stress of a graph* G , denoted by st(G) , is the sum of the stresses of all its vertices.

We define the edge partition of *G* with respect to stress defined as $S=\left\{\left(st\left(v_{i}\right),\mathrm{st}\left(v_{j}\right)\right)|v_{i},v_{j}\in E\left(G\right)\right\}$ and for each (a,b)∈S such that

- Stress based Geometric-Arithmetic index (Yuan et al., 2010)
$$GA^{st}\left(a,b\right)=\underset{\left(a,b\right)\in S}{\sum }\frac{2\sqrt{a\cdot b}}{a+b}$$
- Stress based First Zagreb index (Gutman and Trinajstić, 1973)
$$M_{1}^{st}\left(a,b\right)=\underset{\left(a,b\right)\in S}{\sum }\left(a+b\right)$$
- Stress based Second Zagreb index (Gutman and Trinajstić, 1973)
$$M_{2}^{st}\left(a,b\right)=\underset{\left(a,b\right)\in S}{\sum }\left(a\cdot b\right)$$
- Stress based Forgotten index (Furtula and Gutman, 2015)
$$F^{st}\left(a,b\right)=\underset{\left(a,b\right)\in S}{\sum }\left(a^{2}+b^{2}\right)$$
- Stress based Inverse Sum index (Pattabiraman, 2018)
$$IS^{st}\left(a,b\right)=\underset{\left(a,b\right)\in S}{\sum }\frac{a\cdot b}{a+b}$$
- Stress based Shilpa-Shanmukha index (Zhao et al., 2021)
$$SS^{st}\left(a,b\right)=\underset{\left(a,b\right)\in S}{\sum }\sqrt{\frac{a\cdot b}{a+b}}$$
- Stress based Sombor index (Gutman, 2021)
$$SO^{st}\left(a,b\right)=\underset{\left(a,b\right)\in S}{\sum }\sqrt{a^{2}+b^{2}}$$
- Stress based Reverse Randić index (Randić, 2002)
$$RR^{st}\left(a,b\right)=\underset{\left(a,b\right)\in S}{\sum }\sqrt{a\cdot b}$$

In this paper, we focus on the Hodgkin lymphoma drugs such as Carmustine, Cyclophosphamide, Dacarbazine, Lomustine, Chlorambucil, Procarbazine, Pemigatinib, Doxorubicin, Vinblastine, Bleomycin, Dexamethasone, Etoposide, and Prednisone. The molecular structure of these graphs are depicted in Figure 1. To calculate the stress sum based indices of the graph $G_{i}$ , we employed the stress-based edge partition of $G_{i}$ . These partitions are denoted by $S_{i}$ , which represent the possible edge partitions corresponding to the graph $G_{i}$ , and are listed below:

$$S_{1}=\left\{\left(0,34\right),\left(0,10\right),\left(34,36\right),\left(34,24\right),\left(18,36\right),\left(18,24\right),\left(18,10\right),\left(36,10\right)\right\}$$

$$S_{2}=\left\{\left(0,21\right),\left(33,4\right),\left(33,39\right),\left(33,21\right),\left(32,39\right),\left(32,27\right),\left(1,4\right),\left(1,6\right),\left(6,39\right),\left(21,27\right)\right\}$$

$$S_{3}=\left\{\left(0,13\right),\left(0,58\right),\left(3,11\right),\left(11,19\right),\left(13,24\right),\left(19,58\right),\left(24,57\right),\left(57,58\right)\right\}$$

$$S_{4}=\left\{\left(0,14\right),\left(0,62\right),\left(3,12\right),\left(12,21\right),\left(14,56\right),\left(14,26\right),\left(21,57\right),\left(56,57\right),\left(56,26\right),\left(56,62\right)\right\}$$

$$S_{5}=\left\{\left(0,37\right),\left(0,79\right),\left(0,21\right),\left(37,51\right),\left(40,21\right),\left(40,57\right),\left(41,43\right),\left(41,75\right),\left(43,87\right),\left(75,57\right),\left(79,51\right),\left(79,87\right)\right\}$$

$$\begin{array}{cc} S_{6}= & \left\{\left(0,49\right),\left(0,24\right),\left(65,129\right),\left(65,64\right),\left(129,123\right),\left(64,123\right),\left(69,88\right),\left(69,49\right),\left(88,105\right),\left(105,123\right),\right.\\ & \left.\left(46,24\right),\left(46,123\right)\right\} \end{array}$$

$$\begin{array}{cc} S_{7}= & \left\{\left(0,194\right),\left(0,132\right),\left(0,75\right),\left(0,119\right),\left(0,41\right),\left(0,240\right),\left(0,251\right),\left(65,205\right),\left(65,57\right),\left(194,6\right),\left(194,119\right),\right.\\ & \left.\left(194,251\right),\left(132,110\right),\left(132,250\right),\left(6,19\right),\left(135,85\right),\left(135,240\right),\left(135,57\right),\left(75,85\right),\left(75,55\right),\left(205,250\right),\right.\\ & \left.\left(205,190\right),\left(82,240\right),\left(82,55\right),\left(19,190\right),\left(119,41\right),\left(110,251\right),\left(240,250\right),\left(251,190\right)\right\} \end{array}$$

$$\begin{array}{cc} S_{8}= & \left\{\left(0,324\right),\left(0,134\right),\left(0,267\right),\left(0,147\right),\left(0,277\right),\left(0,86\right),\left(0,287\right),\left(0,224\right),\left(0,291\right),\left(0,40\right),\left(0,46\right),\left(324,147\right),\left(324,277\right),\right.\\ & \left.\left(324,291\right),\left(69,81\right),\left(69,157\right),\left(134,224\right),\left(134,46\right),\left(267,287\right),\left(267,291\right),\left(267,44\right),\right.\left(81,291\right),\left(147,122\right),\\ & \left.\left(277,157\right),\left(277,94\right),\left(86,99\right),\left(86,63\right),\left(157,99\right),\left(94,63\right),\left(287,224\right),\right.\left.\left(287,122\right),\left(224,40\right),\left(40,44\right)\right\} \end{array}$$

$$\begin{array}{cc} S_{9}= & \left\{\left(0,280\right),\left(0,223\right),\left(0,37\right),\left(0,52\right),\left(3,47\right),\left(518,272\right),\left(518,280\right),\left(518,496\right),\left(267,91\right),\left(267,301\right),\right.\\ & \left.\left(333,337\right),\left(333,442\right),\left(272,291\right),\left(337,27\right),\left(337,301\right),\left(83,27\right),\left(83,45\right),\left(83,442\right),\left(21,151\right),\left(151,223\right),\right.\\ & \left.\left(151,52\right),\left(280,304\right),\left(91,47\right),\left(223,496\right),\left(480,291\right),\left(480,304\right),\left(480,442\right),\left(291,56\right),\left(37,304\right),\left(45,56\right)\right\} \end{array}$$

$$\begin{array}{cc} S_{10}= & \left\{\left(0,64\right),\left(0,65\right),\left(0,259\right),\left(0,327\right),\left(0,137\right),\left(0,343\right),\left(0,98\right),\left(0,304\right),\left(0,433\right),\left(0,188\right),\left(64,188\right),\left(65,209\right),\right.\\ & \left.\left(259,221\right),\left(259,368\right),\left(323,209\right),\left(323,343\right),\left(323,221\right),\left(323,213\right),\left(327,157\right),\left(327,188\right),\left(137,98\right),\right.\\ & \left.\left(137,170\right),\left(209,27\right),\left(18,83\right),\left(18,27\right),\left(83,221\right),\left(213,376\right),\left(343,472\right),\left(472,433\right),\left(472,368\right),\left(157,564\right),\right.\\ & \left.\left(415,170\right),\left(415,440\right),\left(376,304\right),\left(376,563\right),\left(304,368\right),\left(433,563\right),\left(563,564\right),\left(564,440\right)\right\} \end{array}$$

$$\begin{array}{cc} S_{11}= & \left\{\left(0,129\right),\left(0,264\right),\left(0,62\right),\left(0,70\right),\left(0,119\right),\left(0,356\right),\left(128,145\right),\left(128,23\right),\left(128,123\right),\left(2,23\right),\left(2,70\right),\right.\\ & \left.\left(387,709\right),\left(387,70\right),\left(387,370\right),\left(129,233\right),\left(1,21\right),\left(1,23\right),\left(264,163\right),\left(264,356\right),\left(137,479\right),\right.\\ & \left.\left(137,123\right),\left(268,233\right),\left(268,631\right),\left(145,496\right),\left(21,123\right),\left(23,370\right),\left(155,203\right),\left(155,109\right),\left(163,115\right),\right.\\ & \left.\left(675,690\right),\left(675,356\right),\left(675,251\right),\left(690,759\right),\left(62,233\right),\left(709,479\right),\left(709,631\right),\left(203,251\right),\left(119,109\right),\right.\\ & \left.\left(119,115\right),\left(479,496\right),\left(759,496\right),\left(759,370\right)\right\} \end{array}$$

$$\begin{array}{cc} S_{12}= & \left\{\left(0,262\right),\left(0,152\right),\left(0,176\right),\left(0,181\right),\left(0,60\right),\left(0,65\right),\left(0,67\right),\left(0,197\right),\left(0,71\right),\left(0,72\right),\left(0,499\right),\left(513,1233\right),\right.\\ & \left.\left(513,475\right),\left(1025,963\right),\left(1025,225\right),\left(1025,994\right),\left(129,12\right),\left(129,46\right),\left(129,455\right),\left(129,500\right),\left(4,13\right),\right.\\ & \left.\left(4,71\right),\left(1,46\right),\left(1,60\right),\left(262,269\right),\left(262,51\right),\left(262,71\right),\left(520,168\right),\left(520,319\right),\left(520,1233\right),\left(267,421\right),\right.\\ & \left.\left(267,181\right),\left(12,48\right),\left(13,75\right),\left(269,475\right),\left(141,167\right),\left(141,168\right),\left(141,71\right),\left(152,475\right),\left(152,225\right),\right.\\ & \left.\left(421,455\right),\left(421,499\right),\left(167,319\right),\left(167,75\right),\left(168,225\right),\left(168,255\right),\left(48,994\right),\left(176,60\right),\left(176,499\right),\right.\\ & \left.\left(51,123\right),\left(60,455\right),\left(319,117\right),\left(65,455\right),\left(67,197\right),\left(963,1150\right),\left(197,1233\right),\left(70,117\right),\left(70,123\right),\right.\\ & \left.\left(72,255\right),\left(455,500\right),\left(1233,1150\right),\left(475,994\right),\left(475,104\right),\left(475,499\right),\left(994,500\right),\left(104,123\right),\left(1150,255\right)\right\} \end{array}$$

$$\begin{array}{cc} S_{13}= & \left\{\left(0,1793\right),\left(0,2339\right),\left(0,334\right),\left(0,2129\right),\left(0,356\right),\left(0,100\right),\left(0,2417\right),\left(0,115\right),\left(0,391\right),\left(0,2207\right),\left(0,197\right),\right.\\ & \left.\left(0,199\right),\left(0,1737\right),\left(0,715\right),\left(0,208\right),\left(0,211\right),\left(0,2277\right),\left(0,743\right),\left(0,232\right),\left(1793,1659\right),\left(1793,2043\right),\right.\\ & \left.\left(1,1\right),\left(1,97\right),\left(1539,1600\right),\left(1539,1477\right),\left(10,820\right),\left(10,88\right),\left(2059,2090\right),\left(2059,2425\right),\left(15,83\right),\right.\\ & \left.\left(15,1205\right),\left(1303,1360\right),\left(1303,441\right),\left(1303,715\right),\left(2331,2339\right),\left(2331,2417\right),\left(2339,2277\right),\left(291,568\right),\right.\\ & \left.\left(291,384\right),\left(291,197\right),\left(294,199\right),\left(294,1018\right),\left(2090,419\right),\left(2090,1722\right),\left(820,880\right),\left(820,743\right),\right.\\ & \left.\left(564,475\right),\left(564,743\right),\left(54,1152\right),\left(54,208\right),\left(568,715\right),\left(568,211\right),\left(570,480\right),\left(570,1018\right),\left(1600,1659\right),\right.\\ & \left.\left(334,1722\right),\left(334,232\right),\left(1360,1722\right),\left(2129,2207\right),\left(2129,2016\right),\left(83,1477\right),\left(88,1132\right),\left(1630,356\right),\right.\\ & \left.\left(1630,1160\right),\left(1630,1737\right),\left(97,385\right),\left(356,208\right),\left(100,379\right),\left(1132,880\right),\left(1132,1205\right),\left(2417,3264\right),\right.\\ & \left.\left(115,378\right),\left(2425,385\right),\left(2425,3264\right),\left(378,419\right),\left(378,232\right),\left(279,441\right),\left(379,211\right),\left(1152,1160\right),\right.\\ & \left.\left(1152,1018\right),\left(384,475\right),\left(391,199\right),\left(391,480\right),\left(2207,2277\right),\left(1205,1245\right),\left(3264,1771\right),\left(197,2043\right),\right.\\ & \left.\left(1477,1245\right),\left(1737,1771\right),\left(2016,2043\right)\right\} \end{array}$$

**FIGURE 1:**
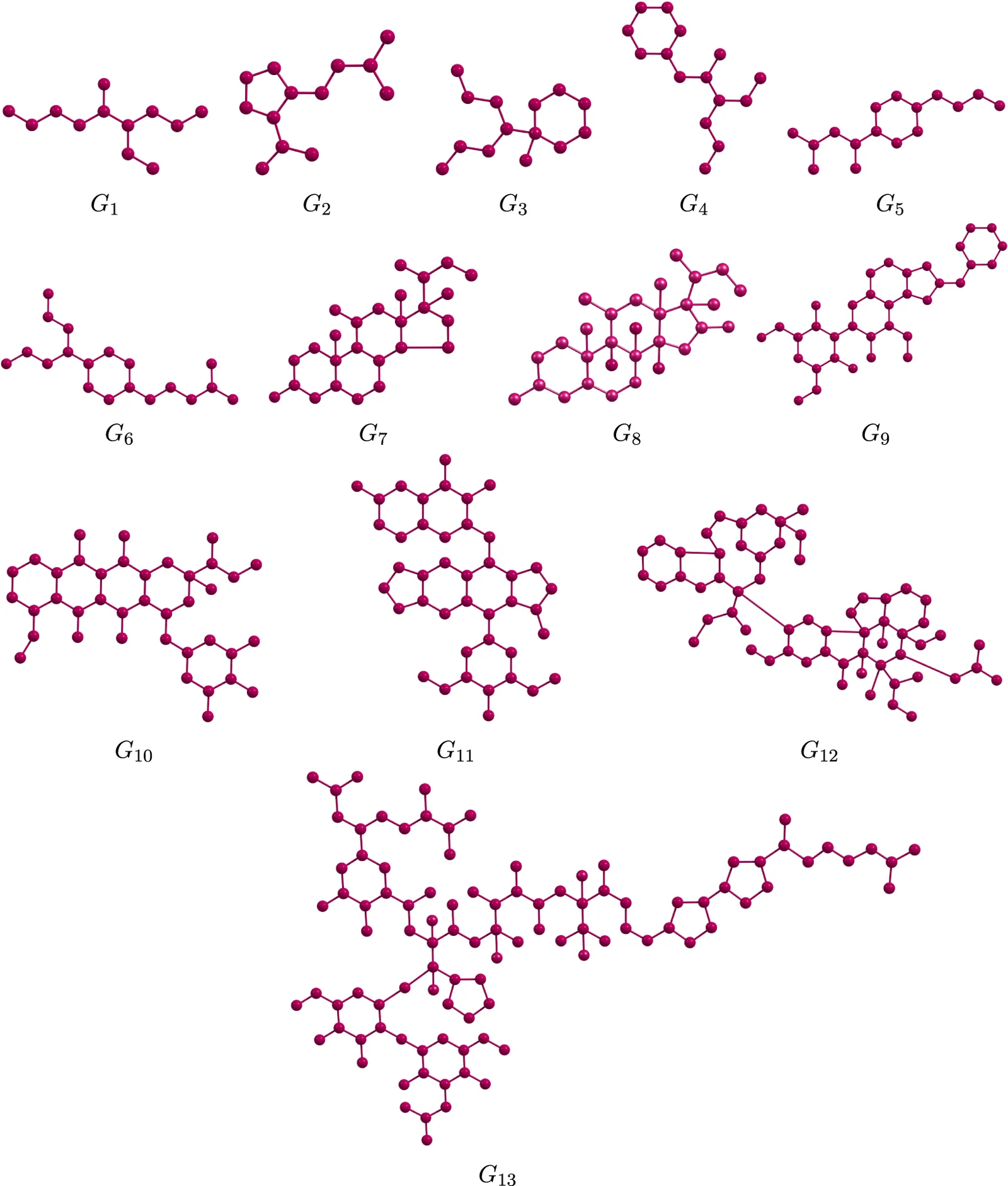
Structures of selected drugs used in Hodgkin lymphoma therapy.

Let ${st}_{G_{i}}\left(S_{i}\right)$ be an array in which each entry equals the number of edges uv having st(u)=a and st(v)=b for each $\left(a,b\right)\in S_{i}$ for $G_{i}$ .

$$\begin{array}{cc} {st}_{G_{1}}\left(S_{1}\right)= & \left[1,3,1,1,1,1,2,1\right]\\ {st}_{G_{2}}\left(S_{2}\right)= & \left[4,1,1,1,1,1,1,1,1,1\right]\\ {st}_{G_{3}}\left(S_{3}\right)= & \left[2,1,2,2,2,2,2,1\right]\\ {st}_{G_{4}}\left(S_{4}\right)= & \left[2,1,2,2,1,1,2,1,1,2\right]\\ {st}_{G_{5}}\left(S_{5}\right)= & \left[2,1,1,1,1,1,2,2,2,1,1,1\right]\\ {st}_{G_{6}}\left(S_{6}\right)= & \left[2,2,2,2,1,2,1,1,1,1,2,2\right]\\ {st}_{G_{7}}\left(S_{7}\right)= & \left[1,1,1,1,1,1,1,1,1,1,1,1,1,1,1,1,1,1,1,1,1,1,1,1,\right.\\ & \;\left.1,1,1,1,1,1\right]\\ {st}_{G_{8}}\left(S_{8}\right)= & \left[1,1,1,1,1,1,1,1,1,1,1,1,1,1,1,1,1,1,1,1,1,1,1,1,\right.\\ & \;\left.1,1,1,1,1,1,1,1,1,1\right]\\ {st}_{G_{9}}\left(S_{9}\right)= & \left[1,2,1,2,2,1,1,1,2,1,1,1,1,1,1,1,1,1,2,2,2,1,2,2,\right.\\ & \;\left.1,1,1,1,1,1,1\right]\\ {st}_{G_{10}}\left(S_{10}\right)= & \left[1,1,1,1,2,1,1,1,1,1,1,1,1,1,1,1,1,1,1,1,2,2,1,1,\right.\\ & \;\left.1,1,1,1,1,1,1,1,1,1,2,1,1,1,1,1,1,1\right]\\ {st}_{G11}\left(S_{11}\right)= & \left[1,1,2,1,1,1,1,1,1,1,1,1,1,1,2,1,1,1,1,1,1,2,2,\right.\\ & \;\left.1,1,1,1,1,1,1,1,1,1,1,2,1,1,1,1,1,1,1,1,1\right]\\ {st}_{G12}\left(S_{12}\right)= & \left[1,1,1,2,1,1,1,1,1,1,1,1,1,1,1,1,1,1,1,1,1,1,1,1,\right.\\ & \;\left.1,1,1,1,1,1,1,1,1,1,1,1,1,1,1,1,1,1,1,1,1,1,1,\right.\\ & \;\left.1,1,1,1,1,1,1,1,1,1,1,1,1\right]\\ {st}_{G_{13}}\left(S_{13}\right)= & \left[1,1,1,1,1,1,1,1,1,1,6,4,1,1,1,1,1,1,1,1,1,2,1,1,\right.\\ & \;\left.1,1,1,1,1,1,1,1,1,1,1,1,1,1,2,1,1,1,1,1,1,1,1,1,\right.\\ & \;\left.1,1,1,1,1,1,1,1,1,1,1,1,1,1,1,1,1,2,1,1,1,1,1,1,\right.\\ & \;\left.1,1,1,1,1,1,1,1,1,1,1,1,1,1,1,1,1,1,1,1,1,1,1,1\right] \end{array}$$

### 2.1 Detailed computation for Carmustine

To illustrate the computation of the proposed stress sum-based topological indices, we consider the molecular graph $G_{1}$ of Carmustine. The stress value st(v) of each vertex $v\in V\left(G_{1}\right)$ is determined by counting the number of shortest paths passing through v . For each edge $uv\in E\left(G_{1}\right)$ , the pair (st(u),st(v)) is then considered. The distinct stress pairs corresponding to the edges of $G_{1}$ form the stress-based edge partition $S_{1}$ .

For the molecular graph of Carmustine, the stress-based edge partition is given by

$$S_{1}=\left\{\left(0,34\right),\left(0,10\right),\left(34,36\right),\left(34,24\right),\left(18,36\right),\left(18,24\right),\left(18,10\right),\left(36,10\right)\right\}.$$

The corresponding number of edges associated with each stress pair in $S_{1}$ is given by

$${st}_{G_{1}}\left(S_{1}\right)=\left[1,3,1,1,1,1,2,1\right].$$

Thus, the pair (0, 34) occurs once, (0, 10) occurs three times, (34, 36), (34, 24), (18, 36), (18, 24), and (36, 10) each occur once, while (18, 10) occurs twice.

The stress-based edge partition of $G_{1}$ and its corresponding multiplicities are summarized in Table 1.

**TABLE 1:** Stress-based edge partition of the molecular graph of Carmustine.

i	Stress pair (a,b)	${st}_{G_{1}}\left(a,b\right)$
1	(0, 34)	1
2	(0, 10)	3
3	(34, 36)	1
4	(34, 24)	1
5	(18, 36)	1
6	(18, 24)	1
7	(18, 10)	2
8	(36, 10)	1

Using the above stress-based edge partition and its multiplicities, the eight proposed indices for $G_{1}$ can be calculated as follows.

#### 2.1.1 Stress-based Geometric–Arithmetic index

The stress-based Geometric–Arithmetic index is given by

$$\begin{array}{ll} GA^{st}\left(G_{1}\right)= & 1\left(\frac{2\sqrt{0\left(34\right)}}{0+34}\right)+3\left(\frac{2\sqrt{0\left(10\right)}}{0+10}\right)+1\left(\frac{2\sqrt{34\left(36\right)}}{34+36}\right)+1\left(\frac{2\sqrt{34\left(24\right)}}{34+24}\right)\\ & \;+1\left(\frac{2\sqrt{18\left(36\right)}}{18+36}\right)+1\left(\frac{2\sqrt{18\left(24\right)}}{18+24}\right)+2\left(\frac{2\sqrt{18\left(10\right)}}{18+10}\right)+1\left(\frac{2\sqrt{36\left(10\right)}}{36+10}\right)\\ = & 6.6587404177\\ \approx & 6.659. \end{array}$$

#### 2.1.2 Stress-based First Zagreb index

The stress-based First Zagreb index is calculated as

$$\begin{array}{ll} M_{1}^{st}\left(G_{1}\right)= & 1\left(0+34\right)+3\left(0+10\right)+1\left(34+36\right)+1\left(34+24\right)\\ & \;+1\left(18+36\right)+1\left(18+24\right)+2\left(18+10\right)+1\left(36+10\right)\\ = & 34+30+70+58+54+42+56+46\\ = & 390. \end{array}$$

#### 2.1.3 Stress-based Second Zagreb index

Similarly, the stress-based Second Zagreb index is

$$\begin{array}{ll} M_{2}^{st}\left(G_{1}\right)= & 1\left(0\cdot 34\right)+3\left(0\cdot 10\right)+1\left(34\cdot 36\right)+1\left(34\cdot 24\right)\\ & \;+1\left(18\cdot 36\right)+1\left(18\cdot 24\right)+2\left(18\cdot 10\right)+1\left(36\cdot 10\right)\\ = & 0+0+1224+816+648+432+360+360\\ = & 3840. \end{array}$$

#### 2.1.4 Stress-based Forgotten index

The stress-based Forgotten index is given by

$$\begin{array}{ll} F^{st}\left(G_{1}\right)= & 1\left(0^{2}+34^{2}\right)+3\left(0^{2}+10^{2}\right)+1\left(34^{2}+36^{2}\right)+1\left(34^{2}+24^{2}\right)\\ & \;+1\left(18^{2}+36^{2}\right)+1\left(18^{2}+24^{2}\right)+2\left(18^{2}+10^{2}\right)+1\left(36^{2}+10^{2}\right)\\ = & 1156+300+2452+1732\\ & \;+1620+900+848+1396\\ = & 10404. \end{array}$$

#### 2.1.5 Stress-based Inverse Sum index

The stress-based Inverse Sum index is calculated as

$$\begin{array}{ll} IS^{st}\left(G_{1}\right)= & 1\left(\frac{0\cdot 34}{0+34}\right)+3\left(\frac{0\cdot 10}{0+10}\right)+1\left(\frac{34\cdot 36}{34+36}\right)+1\left(\frac{34\cdot 24}{34+24}\right)\\ & \;+1\left(\frac{18\cdot 36}{18+36}\right)+1\left(\frac{18\cdot 24}{18+24}\right)+2\left(\frac{18\cdot 10}{18+10}\right)+1\left(\frac{36\cdot 10}{36+10}\right)\\ = & 74.5236239023\\ \approx & 74.524. \end{array}$$

#### 2.1.6 Stress-based Shilpa–Shanmukha index

The stress-based Shilpa–Shanmukha index is

$$\begin{array}{ll} SS^{st}\left(G_{1}\right)= & 1\sqrt{\frac{0\cdot 34}{0+34}}+3\sqrt{\frac{0\cdot 10}{0+10}}+1\sqrt{\frac{34\cdot 36}{34+36}}+1\sqrt{\frac{34\cdot 24}{34+24}}\\ & \;+1\sqrt{\frac{18\cdot 36}{18+36}}+1\sqrt{\frac{18\cdot 24}{18+24}}+2\sqrt{\frac{18\cdot 10}{18+10}}+1\sqrt{\frac{36\cdot 10}{36+10}}\\ = & 22.4721307557\\ \approx & 22.472. \end{array}$$

#### 2.1.7 Stress-based Sombor index

The stress-based Sombor index is calculated as

$$\begin{array}{ll} SO^{st}\left(G_{1}\right)= & 1\sqrt{0^{2}+34^{2}}+3\sqrt{0^{2}+10^{2}}+1\sqrt{34^{2}+36^{2}}+1\sqrt{34^{2}+24^{2}}\\ & \;+1\sqrt{18^{2}+36^{2}}+1\sqrt{18^{2}+24^{2}}+2\sqrt{18^{2}+10^{2}}+1\sqrt{36^{2}+10^{2}}\\ = & 303.9298052494\\ \approx & 303.930. \end{array}$$

#### 2.1.8 Stress-based Reverse Randić index

Finally, the stress-based Reverse Randić index is

$$\begin{array}{ll} RR^{st}\left(G_{1}\right)= & 1\sqrt{0\cdot 34}+3\sqrt{0\cdot 10}+1\sqrt{34\cdot 36}+1\sqrt{34\cdot 24}\\ & \;+1\sqrt{18\cdot 36}+1\sqrt{18\cdot 24}+2\sqrt{18\cdot 10}+1\sqrt{36\cdot 10}\\ = & 155.5983605878\\ \approx & 155.598. \end{array}$$

Therefore, the eight stress sum-based topological indices obtained for the molecular graph $G_{1}$ of Carmustine are

$$\begin{array}{cccc} GA^{st}\left(G_{1}\right) & =6.659, & M_{1}^{st}\left(G_{1}\right) & =390,\\ M_{2}^{st}\left(G_{1}\right) & =3840, & F^{st}\left(G_{1}\right) & =10404,\\ IS^{st}\left(G_{1}\right) & =74.524, & SS^{st}\left(G_{1}\right) & =22.472,\\ SO^{st}\left(G_{1}\right) & =303.930, & RR^{st}\left(G_{1}\right) & =155.598. \end{array}$$

These values agree with the corresponding entries reported in Table 2. The same procedure is followed for the remaining twelve Hodgkin lymphoma drug molecular graphs.

**TABLE 2:** Stress sum based topological indices.

Drug	Molecular graph	GA	$M_{1}$	$M_{2}$	F	IS	SS	SO	RR
Carmustine	$G_{1}$	6.659	390	3840	10404	74.524	22.472	303.930	155.598
Dacarbazine	$G_{2}$	7.756	482	5035	14088	85.167	25.256	383.632	181.968
Cyclophosphamide	$G_{3}$	10.029	677	9354	28129	126.661	35.304	532.381	273.593
Lomustine	$G_{4}$	10.980	883	15710	41453	175.916	43.201	680.596	372.526
Procarbazine	$G_{5}$	11.682	1508	37342	92314	316.048	61.071	1141.441	649.142
Chlorambucil	$G_{6}$	14.436	2592	101833	239012	568.446	90.950	1934.476	1178.279
Prednisone	$G_{7}$	19.997	7098	457857	1298380	1330.003	161.953	5562.068	2805.385
Dexamethasone	$G_{8}$	20.591	9608	706787	2206238	1669.925	184.534	7682.120	3521.563
Pemigatinib	$G_{9}$	28.710	15688	2095970	5216366	3209.783	296.828	11990.788	6840.734
Doxorubicin	$G_{10}$	30.306	20352	2989350	7255616	4201.802	349.583	15480.308	8679.096
Etoposide	$G_{11}$	34.942	23879	4562521	10897985	5097.394	406.273	17994.597	10722.752
Vinbastine	$G_{12}$	47.929	40959	10094837	27117891	8070.513	594.289	31708.853	17542.919
Bleomycin	$G_{13}$	65.179	171187	113768961	289100329	34048.948	1402.078	131634.261	70811.807

## 3 QSPR analysis

Quantitative structure–property relationship modeling is a key tool in drug discovery, linking molecular structure to physicochemical behavior. We analyzed thirteen drug-like molecules relevant to Hodgkin lymphoma, considering properties such as boiling point (BP), complexity (C), heavy atom count (HAC), molecular weight (MW), molar refractivity (MR), polarizability (P), and polar surface area (PSA). These properties influence absorption, distribution, metabolism, and overall pharmacokinetics.

Data were obtained from ChemSpider and PubChem, and are summarized in Table 3. Molecular structures were visualized using Chemcraft (Figure 1), and statistical analyses were performed with SageMath 9.3.

**TABLE 3:** Physicochemical properties of HL-related drugs.

Drug	Physicochemical properties						
	Boiling point ( ° C)	Complexity	Heavy atom count	Molar refraction ( ${\text{cm}}^{3}$ /mol)	Molecular weight (g/mol)	Polar surface area	Polarizability
Carmustine	309.6	156	12	46.6	214.05	65	18.5
Dacarbazine	456.3	215	13	46.2	182.18	100	18.3
Cyclophosphamide	336.1	212	14	58.1	261.08	51	23
Lomustine	-	219	15	57.8	233.69	62	22.9
Procarbazine	384.6	210	16	65.8	221.3	53	26.1
Chlorambucil	460	250	19	79.9	304.2	41	31.7
Prednisone	573.7	764	26	94.1	358.4	92	37.3
Dexamethasone	568.2	805	28	100.2	395.2	95	39.7
Pemigatinib	-	731	35	125.1	487.5	83	49.6
Doxorubicin	-	977	39	-	543.5	-	-
Etoposide	798.1	969	42	140.1	588.6	161	55.5
Vinblastine	-	1700	59	220.7	811	154	87.5
Bleomycin	-	2580	96	-	1415.6	684	-

Linear regression was used to model the relationship between topological indices and physicochemical properties:

Q=αT+β

where Q is the property, T the topological index, α the slope, and β the intercept. Correlation heat map is shown in Figure 2, $R^{2}$ values are given in Table 2, and QSPR models with corresponding $R^{2}$ , RMSE, and significance values are listed in Table 4. The coefficient of determination $\left(r^{2}\right)$ measures how well the model explains variance, with higher values indicating stronger predictive power. The adjusted $r^{2}$ refines this by accounting for the number of predictors, ensuring model accuracy. The root mean square error (RMSE) indicates the average magnitude of prediction errors, with lower values representing better model fit, while the F-value determines overall model significance. Table 5 displays the best fit with statistical parameters such as $r^{2}$ , RMSE, F-stat and p-value. This framework quantifies how molecular structure, represented by topological indices, influences key physicochemical characteristics.

**FIGURE 2:**
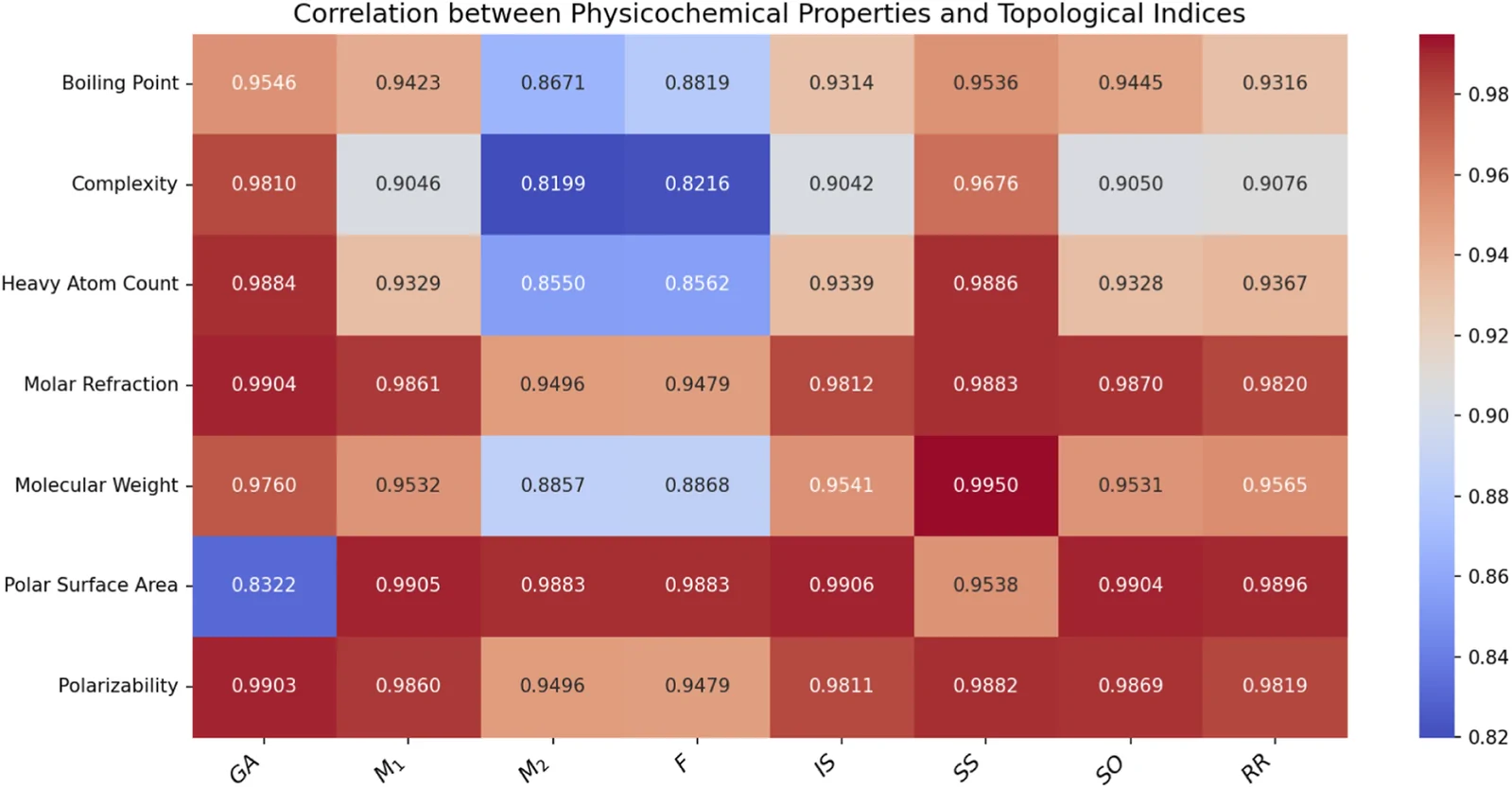
Correlation heatmap between physicochemical properties and topological indices of Hodgkin lymphoma drugs.

**TABLE 4:** $R^{2}$ value for stress sum based topological indices.

Topological indices	BP	C	HAC	MR	MW	PSA	P
GA	**0.9113**	**0.9624**	0.9770	**0.9808**	0.9525	0.6926	**0.9806**
$M_{1}$	0.8879	0.8183	0.8702	0.9723	0.9085	0.9811	0.9722
$M_{2}$	0.7520	0.6722	0.7309	0.9018	0.7844	0.9768	0.9018
F	0.7779	0.6751	0.7330	0.8987	0.7863	0.9767	0.8987
IS	0.8675	0.8176	0.8721	0.9627	0.9103	**0.9814**	0.9625
SS	0.9093	0.9363	**0.9773**	0.9767	**0.9900**	0.9097	0.9765
SO	0.8921	0.8190	0.8701	0.9741	0.9084	0.9808	0.9740
RR	0.8678	0.8238	0.8775	0.9643	0.9148	0.9794	0.9642

R^2^ value for stress sum based topological indices (maximum value of R^2^ for each of the physicochemical properties is highlighted).

**TABLE 5:** Best fit linear regression models for physicochemical properties of drug structures.

Properties	Regression equation	N	adj $\left(r^{2}\right)$	$r^{2}$	RMSE	F	p
BP	$BP=16.309\left(GA^{\text{st}}\right)+228.77$	8	0.8965	0.9113	51.20	61.62	0.00
C	$C=40.128\left(GA^{\text{st}}\right)-201.49$	13	0.9590	0.9624	144.52	281.36	0.00
HAC	$HAC=0.0621\left(SS^{\text{st}}\right)+14.301$	13	0.9753	0.9773	3.75	474.62	0.00
MR	$MR=3.9872\left(GA^{\text{st}}\right)+16.59$	11	0.9787	0.9808	7.63	459.60	0.00
MW	$MW=0.8895\left(SS^{\text{st}}\right)+211.42$	13	0.9891	0.9900	35.42	1091.61	0.00
PSA	$PSA=0.0182\left(IS^{\text{st}}\right)+53.705$	12	0.9795	0.9814	25.27	526.67	0.00
P	$P=1.5805\left(GA^{\text{st}}\right)+6.5763$	11	0.9785	0.9806	3.04	455.30	0.00

### 3.1 Linear regression models

To examine the relationship between the proposed stress sum-based topological indices and the selected physicochemical properties, simple linear regression models were developed for each pair of topological index and physicochemical property. In total, 56 linear regression models were obtained by considering eight topological indices and seven physicochemical properties. The corresponding regression equations are presented below.

For the stress-based Geometric–Arithmetic index $GA^{st}$ , the regression equations are given by

$$\begin{array}{ll} BP & =16.309390\left(GA^{st}\right)+228.767694,\\ C & =40.127934\left(GA^{st}\right)-201.489579,\\ HAC & =1.352545\left(GA^{st}\right)-0.323112,\\ MR & =3.987249\left(GA^{st}\right)+16.589607,\\ MW & =19.011349\left(GA^{st}\right)+10.621707,\\ PSA & =8.114869\left(GA^{st}\right)-51.845681,\\ P & =1.580463\left(GA^{st}\right)+6.576320. \end{array}$$

For the stress-based First Zagreb index $M_{1}^{st}$ , the corresponding regression equations are

$$\begin{array}{ll} BP & =0.018589\left(M_{1}^{st}\right)+378.393491,\\ C & =0.013955\left(M_{1}^{st}\right)+435.924658,\\ HAC & =0.000481\left(M_{1}^{st}\right)+20.910306,\\ MR & =0.003989\left(M_{1}^{st}\right)+56.423059,\\ MW & =0.007002\left(M_{1}^{st}\right)+303.730954,\\ PSA & =0.003620\left(M_{1}^{st}\right)+53.806718,\\ P & =0.001581\left(M_{1}^{st}\right)+22.365105. \end{array}$$

For the stress-based Second Zagreb index $M_{2}^{st}$ , the regression equations are

$$\begin{array}{ll} BP & =0.000088\left(M_{2}^{st}\right)+421.087219,\\ C & =0.000019\left(M_{2}^{st}\right)+558.462887,\\ HAC & =0.000001\left(M_{2}^{st}\right)+25.062502,\\ MR & =0.000016\left(M_{2}^{st}\right)+67.994703,\\ MW & =0.000010\left(M_{2}^{st}\right)+362.756101,\\ PSA & =0.000005\left(M_{2}^{st}\right)+77.785014,\\ P & =0.000006\left(M_{2}^{st}\right)+26.951363. \end{array}$$

For the stress-based Forgotten index $F^{st}$ , the regression equations are

$$\begin{array}{ll} BP & =0.000037\left(F^{st}\right)+416.514363,\\ C & =0.000007\left(F^{st}\right)+557.594877,\\ HAC & =0.000000\left(F^{st}\right)+25.037093,\\ MR & =0.000006\left(F^{st}\right)+68.408316,\\ MW & =0.000004\left(F^{st}\right)+362.401097,\\ PSA & =0.000002\left(F^{st}\right)+77.574977,\\ P & =0.000002\left(F^{st}\right)+27.115101. \end{array}$$

For the stress-based Inverse Sum index $IS^{st}$ , the regression equations are

$$\begin{array}{ll} BP & =0.087134\left(IS^{st}\right)+384.878567,\\ C & =0.070103\left(IS^{st}\right)+434.898639,\\ HAC & =0.002422\left(IS^{st}\right)+20.858075,\\ MR & =0.019747\left(IS^{st}\right)+56.849705,\\ MW & =0.035225\left(IS^{st}\right)+302.990612,\\ PSA & =0.018194\left(IS^{st}\right)+53.705174,\\ P & =0.007828\left(IS^{st}\right)+22.534379. \end{array}$$

For the stress-based Shilpa–Shanmukha index $SS^{st}$ , the regression equations are

$$\begin{array}{ll} BP & =1.170018\left(SS^{st}\right)+341.355315,\\ C & =1.816525\left(SS^{st}\right)+239.574430,\\ HAC & =0.062086\left(SS^{st}\right)+14.300829,\\ MR & =0.277237\left(SS^{st}\right)+45.610490,\\ MW & =0.889512\left(SS^{st}\right)+211.416905,\\ PSA & =0.424729\left(SS^{st}\right)+19.092744,\\ P & =0.109892\left(SS^{st}\right)+18.079408. \end{array}$$

For the stress-based Sombor index $SO^{st}$ , the regression equations are

$$\begin{array}{ll} BP & =0.024593\left(SO^{st}\right)+376.586819,\\ C & =0.018157\left(SO^{st}\right)+435.841017,\\ HAC & =0.000626\left(SO^{st}\right)+20.913138,\\ MR & =0.005188\left(SO^{st}\right)+56.365048,\\ MW & =0.009106\left(SO^{st}\right)+303.775105,\\ PSA & =0.004707\left(SO^{st}\right)+53.768800,\\ P & =0.002056\left(SO^{st}\right)+22.342055. \end{array}$$

Finally, for the stress-based Reverse Randić index $RR^{st}$ , the regression equations are

$$\begin{array}{ll} BP & =0.041410\left(RR^{st}\right)+384.948981,\\ C & =0.033797\left(RR^{st}\right)+431.236008,\\ HAC & =0.001167\left(RR^{st}\right)+20.739651,\\ MR & =0.009159\left(RR^{st}\right)+57.215293,\\ MW & =0.016961\left(RR^{st}\right)+301.356656,\\ PSA & =0.008730\left(RR^{st}\right)+53.047965,\\ P & =0.003630\left(RR^{st}\right)+22.679166. \end{array}$$

## 4 Results and discussion

The calculation of stress sum-based indices provides a useful means of interpreting the physicochemical behavior of drug molecules. In this work, strong linear relationships were observed between specific indices and selected molecular properties. The geometric–arithmetic index $\left(GA^{st}\right)$ showed high predictive ability for boiling point, complexity, molar refraction, and polarizability, reflecting its ability to describe overall molecular features. Similarly, the Shilpa–Shanmukha index $\left(SS^{st}\right)$ showed strong correlations with molecular weight and heavy atom count, highlighting its effectiveness in capturing additive atomic contributions. The inverse sum indeg index $\left(IS^{st}\right)$ was closely associated with polar surface area, indicating its sensitivity to molecular structural and surface characteristics. The corresponding best-fit linear regression equations and their statistical parameters are presented in Table 5. As shown in Table 5, the selected models exhibit high $r^{2}$ and adjusted $r^{2}$ values together with low RMSE values, indicating strong linear relationships between the selected topological indices and physicochemical properties. The corresponding scatter plots are presented in Figure 3, which illustrate the linear mapping between the selected stress sum-based topological indices and their respective physicochemical properties.

**FIGURE 3:**
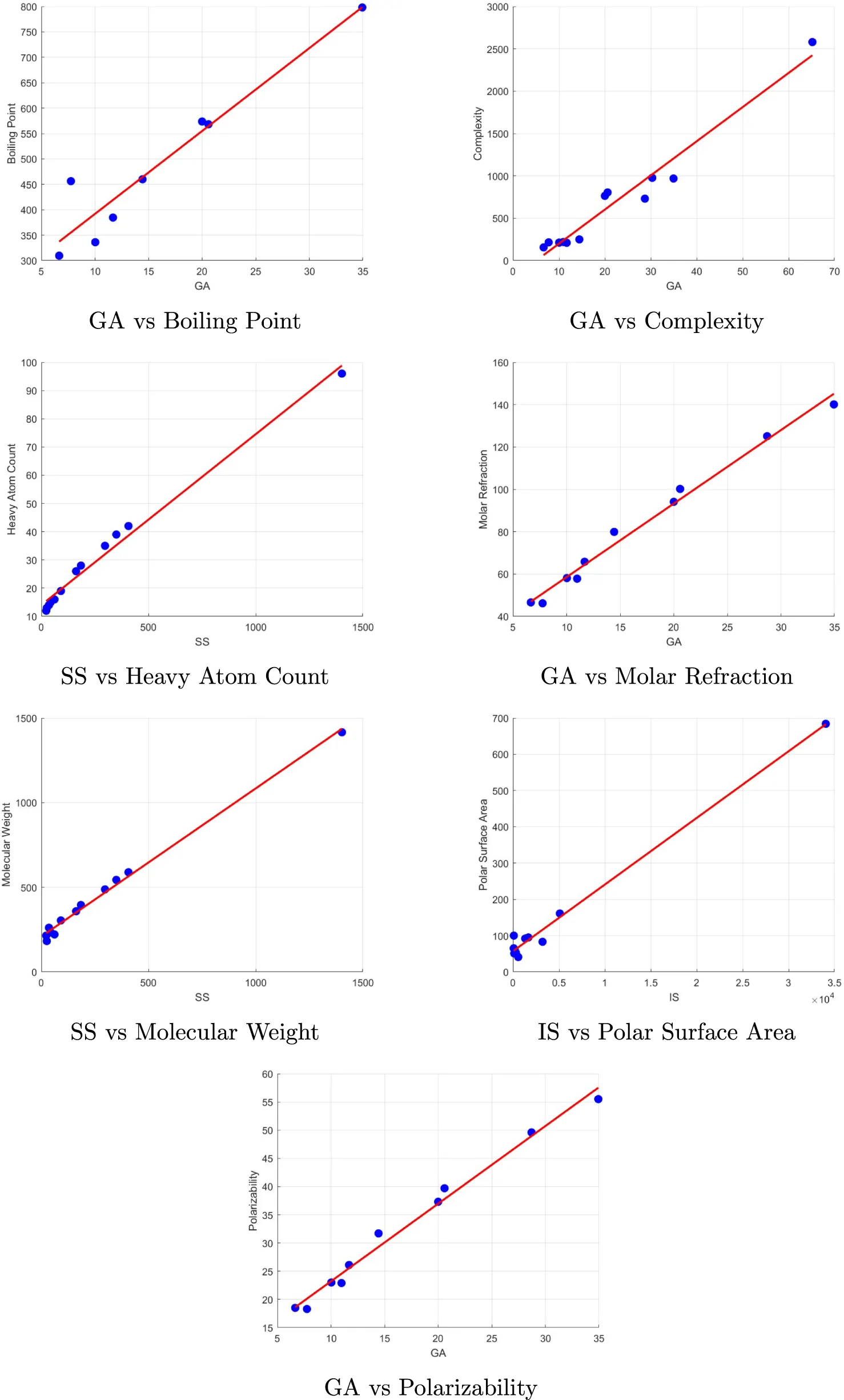
Scatter plots comparing the models of physicochemical properties and topological indices.

## 5 Validation

To evaluate the robustness and predictive reliability of the developed models, validation was performed using leave-one-out cross-validation (LOO-CV). The LOO-CV approach is particularly suitable for relatively small datasets, as each observation is systematically excluded once from the training process and subsequently predicted using a model constructed from the remaining observations. This procedure provides an effective assessment of the model’s stability and its ability to generalize to unseen data.

### 5.1 Leave-one-out cross-validation

The predictive performance of the developed models was assessed using the leave-one-out cross-validation coefficient $\left(Q_{\text{LOO}}^{2}\right)$ . In this method, one compound is removed from the dataset at a time, the model is rebuilt using the remaining (n−1) compounds, and the excluded compound is then predicted. This process is repeated for all compounds in the dataset.

A higher $Q_{\text{LOO}}^{2}$ value indicates superior internal predictive capability and demonstrates the robustness and reliability of the model. In addition to $Q_{\text{LOO}}^{2}$ , the root mean square error of cross-validation (RMSECV) and mean absolute error (MAE) were also calculated to further evaluate prediction accuracy.

The mathematical expression for the leave-one-out cross-validation coefficient is given as:

$$Q_{\text{LOO}}^{2}=1-\frac{\sum_{j=1}^{n}{\left(y_{j}^{\text{act}}-y_{j}^{\text{pred,LOO}}\right)}^{2}}{\sum_{j=1}^{n}{\left(y_{j}^{\text{act}}-{\bar{y}}^{\text{act}}\right)}^{2}}$$

where $y_{j}^{\text{act}}$ represents the experimental value of the $j^{\text{th}}$ compound, $y_{j}^{\text{pred,LOO}}$ denotes the predicted value obtained when the compound is excluded from the training set, and ${\bar{y}}^{\text{act}}$ is the mean of the experimental values. The leave-one-out cross-validation analysis was performed using a Python script developed for this study. The script was used to generate the LOO predictions and calculate $Q_{\text{LOO}}^{2}$ , RMSECV, and MAE for each model. The obtained values were checked to ensure the consistency of the reported results. For transparency and reproducibility, the Python code used for the analysis is publicly available in the authors’ GitHub repository: https://github.com/BharaniDharanKumaravel/Code-for-Graph-problems/blob/df8e97ba147b820294ca6c78bcab13450aa134a8/QSPR_analysis_Leave%20One%20Out%20Cross%20Validation.

The calculated values of $Q_{\text{LOO}}^{2}$ , RMSECV, and MAE are presented in Table 6. Higher $Q_{\text{LOO}}^{2}$ values together with lower RMSECV and MAE values generally indicate better predictive performance and model reliability. As observed from Table 6, the $Q_{\text{LOO}}^{2}$ values range from 0.8558 to 0.9553, indicating a satisfactory predictive performance across the considered physicochemical properties. Among the properties, molecular weight exhibits the highest $Q_{\text{LOO}}^{2}$ value of 0.9553, whereas boiling point shows the lowest value of 0.8558. The corresponding RMSECV and MAE values further provide an assessment of the prediction errors associated with each model.

**TABLE 6:** Statistical parameters $Q_{\text{LOO}}^{2}$ , RMSECV, and MAE obtained from leave-one-out cross-validation.

Properties	$Q_{\text{LOO}}^{2}$	RMSECV	MAE
BP	0.8558	56.5285	38.9673
C	0.9372	171.7356	137.1100
MR	0.9453	11.6520	7.4714
P	0.9447	4.6439	2.9773
HAC	0.8749	8.1109	4.9305
MW	0.9553	69.0112	42.6982
PSA	0.8850	57.3093	34.0628

## 6 Conclusion

This study examined the usefulness of stress sum–based topological indices as molecular descriptors for predicting the physicochemical properties of drugs used for the treatment of Hodgkin lymphoma disease. The linear regression results showed that several of the proposed indices have strong relationships with the selected properties, indicating that stress-based information can provide useful structural information for QSPR analysis. The leave-one-out cross-validation results also showed reasonable predictive performance for several of the developed models, supporting the applicability of the proposed descriptors.

However, some limitations should be noted. In particular, there is currently no standard and widely adopted computational procedure specifically designed for calculating the stress of a vertex in molecular graphs. The calculation of stress values can therefore become computationally demanding as the size and complexity of the molecular graph increase. In addition, the present analysis is based on a relatively small set of thirteen drug molecules, and the regression models are restricted to seven physicochemical properties. Thus, the results should be interpreted within the scope of the molecular dataset considered.

Future work could involve the creation of efficient algorithms for calculating vertex stress in larger molecular graphs and the expansion of the proposed indices to a more diverse set of chemical data. Moreover, a more comprehensive assessment of their predictive ability can be carried out by testing these descriptors on independent data sets and comparing them with other popular molecular descriptors. To summarize, the present work is an application of stress sum-based topological indices in QSPR modeling and their potential for future work in chemical graph theory and molecular property prediction.

## Data Availability

The original contributions presented in the study are included in the article/supplementary material, further inquiries can be directed to the corresponding author.
